# HEALPix-IA: A Global Registration Algorithm for Initial Alignment

**DOI:** 10.3390/s19020427

**Published:** 2019-01-21

**Authors:** Yongzhuo Gao, Zhijiang Du, Wei Xu, Mingyang Li, Wei Dong

**Affiliations:** State Key Laboratory of Robotics and System, Harbin Institute of Technology, Harbin 150001, China; gaoyongzhuo@hit.edu.cn (Y.G.); duzj01@hit.edu.cn (Z.D.); wxu@hit.edu.cn (W.X.); limingyang@hit.edu.cn (M.L.)

**Keywords:** global registration, 3D alignment, point cloud registration, machining allowance analysis

## Abstract

Methods of point cloud registration based on ICP algorithm are always limited by convergence rate, which is related to initial guess. A good initial alignment transformation can sharply reduce convergence time and raise efficiency. In this paper, we propose a global registration method to estimate the initial alignment transformation based on HEALPix (**H**ierarchical **E**qual **A**rea iso**L**atitude **P**ixelation of a sphere), an algorithm for spherical projections. We adopt EGI (Extended Gaussian Image) method to map the normals of the point cloud and estimate the transformation with optimized point correspondence. Cross-correlation method is used to search the best alignment results in consideration of the accuracy and robustness of the algorithm. The efficiency and accuracy of the proposed algorithm were verified with created model and real data from various sensors in comparison with similar methods.

## 1. Introduction

A point cloud is a set of data points in space. A 3D point cloud is generally produced by sensors such as 3D scanner, 3D camera, Light Detection and Ranging equipment (LiDAR) and so on. Although accuracy and density from the sensors above are different [1], similar analysis methods, such as conversion to 3D surfaces, alignment and registration, are processing in different fields. 3D point cloud registration or alignment is a fundamental problem in computer and robotic vision. The main task is to find the transformation describing rigid motion in SE(3) (Lie group for 3D Rigid transformations) between two given sets of the same or similar point clouds through the analysis of local or global feature. This technique is widely applied to object pose estimation [2], simultaneous localization and mapping (SLAM) [3], medical image processing [4], industrial manufacturing and inspection [5,6], culture heritage reconstruction [7,8,9], topography change detection [10], etc. With the development of 3D scanning sensors and reconstruction technology, the accuracy of point cloud data has improved while the cost of scanning sensor has dropped. The point cloud registration technique is gradually entering low cost application fields, especially industrial areas. For the production mode of small lots and multiple categories, it is important to apply industrial robots and other flexible manufacturing equipments into grinding and polishing in consideration of poor working condition, increasing human cost and frequent product changes. However, the robot system needs a whole process with information such as CAD model, machining allowance (MA), tool path planning and process, and process inspection. Among these processes, 3D point cloud registration technique can be applied to MA analysis and process inspection. When a workpiece is scanned, point cloud data are acquired to align with the CAD model. The redundant parts are calculated as machining allowance, which is taken as input for tool path planning and process. On the other hand, registration is applied to calculate the error between workpiece and CAD model in process inspection.

The application mentioned above in industrial field has used coordinate measuring machine (CMM) to acquire sparse patterns, which are used for localization and MA analysis in early studies [5,11,12], and even is still the mainstream method today. Nevertheless, the CMM method is limited by shape of the workpiece. Complex curve surfaces need plenty of time for measuring and recording or even cannot be dealt with. With the development of laser scanners, stereo cameras and other same type of sensors, the density of points is sharply increasing, as we call the dataset “point cloud”. The improved accuracy of points and reconstruction makes it more precise for registration, but more complex. Researchers have proposed different methods for MA registration with dense point cloud. Li, X. et al. [13] introduced a novel two-step rough–precise automatic method. A PCA-based (principal component analysis) rough alignment algorithm is utilized with the precise registration method, which only considers plane feature matching. Wang, H. et al. [14] introduced a heuristic algorithm into PCA/ICP registration method with multi-objective optimization considering envelopment, wall thickness and plane alignment. Sun, Y. et al. [15] combined the multipliers method and the BFGS algorithm to handle many constraints. The objective function is driven by Euclidean oriented distance with the assumption of a small initial rigid transformation and only envelopment constraint is considered. Whatever the optimization constraints, the methods above have been processed by two steps, rough alignment and optimal registration. Since dense point cloud data are acquired, Sac-IA [16], PCA [17,18,19], 3D-NDT [20] and EGI-based alignment algorithms [21,22,23,24,25] are common methods for rough alignment in different applications. Optimal registration methods are always based on ICP algorithm with different process requirements. Generally, the optimal registration algorithms mentioned above are all local methods, which could be trapped into local minima easily [26]. Thus, a good initial transformation could accelerate the convergence and improve accuracy of the optimization method significantly.

We propose a fast global registration method based on EGI mapping to resolve the contradiction between efficiency and accuracy of dense point cloud registration. The main algorithm includes several steps: (1) equal-area pixelization; (2) EGI mapping; (3) point correspondence searching; (4) transformation estimating; and (5) correlation optimization. The point correspondence searching and correlation optimization could achieve a high accuracy within several iterations in our experiments. The proposed method was compared with other initial alignment registration methods, such as Sac-IA, PCA, 3D-NDT and EGI-based methods. Several contributions are made in this paper: First, a new division method is suggested to improve the division efficiency. Second, a point corresponding optimization is proposed to improve the accuracy. Finally, the application of the algorithm to MA analysis is presented.

The rest of the paper is organized as follows: First, the related works on other initial alignment registration methods are introduced in Section 2. The problem is expressed in Section 3. The main strategy, HEALPix-IA Algorithm, is discussed in Section 4. In Section 5, experiments are presented to demonstrate the advantages of this algorithm over other rough registration methods. Section 6 concludes the paper.

## 2. Related Works

Various rough registration methods are suggested for initial alignment to solve the local minima problem within a couple of seconds or even more quickly.

PCA-based algorithm can perform object alignment in real-time and without constraints on the three registration parameters (i.e., translation, rotation, and scaling). Principal Components Analysis (PCA) computes the mutually orthogonal directions of maximum variance in a collection of d-dimensional data and measures the global features by eigenvalues. It is widely used for image registration [27,28] and 3D alignment [17,18,19]. However, PCA-based methods are sensitive to noise [29], which limits accuracy.

The Sample Consensus-Initial Alignment (SAC-IA) algorithm was introduced by Rusu et al. [16,30], using 16-dimensional point feature histograms (FPFH) that describe the local surface structure. FPFH gives a good discriminative power for point correspondence search. Nevertheless, the robustness to outliers, invariance to pose, sampling density and measurement noise are not mentioned and the extensive computational steps result in time-consuming process.

Another 3D point set registration algorithm is the normal distributions transform (NDT) [20] that represents the underlying scene geometry as a Gaussian probability distribution. 3D-NDT method generates disjoint voxels in space and represents points within the voxels as a probability density function (PDF). Its benefit is to give piece-wise smooth spatial representations; however, the division results in discontinuities in the cost function that could be trapped in local minima [31].

EGI-based algorithms are another branch of 3D point registration, which describes the global features by Extended Gaussian Images (EGI) of laser scan. The intensity image mapping from normals is invariant to translation, thus such algorithms have the advantage that pose parameters can be recovered in two separate steps. Latitude and longitude tessellation [21], regular polyhedron tessellation [22], and other complex EGI division methods [23,24,25] have been suggested to raise the accuracy. A voting/correlation procedure, such as spherical correlation, cross correlation and kernel correlation [32] is generated to search the best alignment. However, the accuracy and efficiency of these algorithms are limited by the division method [33] and it is not sensitive to constant EGI (such as a sphere) [34].

In this paper, an EGI-based registration algorithm is introduced with new division method, which is equal area pixelization. We suggest a special procedure for point correspondence optimization to reduce the error introduced by low level division method. In addition, several conditions are configured to avoid the mismatch caused by constant EGI.

## 3. Problem Formulation

In the standard point-to-point ICP algorithm, registration problem is defined with two 3D point-sets $X=\{x_{i}\},i=1,\ldots ,N$ and $Y=\{y_{j}\},j=1,\ldots ,M$, where $x_{i},y_{i}\in R$ are point coordinates. We call X *model* point cloud and Y *data*. The registration problem is to estimate a rigid motion transformation with translation $t\in R^{3}$ and rotation R∈SO(3) (Lie group for 3D rotations), which minimizes the error *E*:

(1) $$E\left(R,t\right)=\sum_{i=1}^{N}e_{i}{\left(R,t\right)}^{2}=\sum_{i=1}^{N}∥Rx_{i}+t-y_{j^{*}}{∥}^{2},$$

where $e_{i}{\left(R,t\right)}^{2}$ is the per-point residual error for $x_{i}$, and ∥·∥ denotes the Euclidean norm. Given r and t, the point $y_{j^{*}}\in Y$ is denoted as the optimal correspondence of $x_{i}$, which is the closest point to the transformed $x_{i}$ in Y, i.e.,

(2) $$j^{*}=\underset{j\in \{1,\ldots ,M\}}{\arg\min}∥Rx_{i}+t-y_{j^{*}}∥.$$

According to ICP algorithm, two prominent cost functions are used to measure “resemblance”: Hausdorff distance and root mean square distances (RMS) [32]. In this paper, we discuss the registration application that *data* and *model* are not totally resembled because of machining allowance. Hausdorff distance shows the measure of maximal error and RMS, which is more suitable for the “under-resembled” registration problem, shows the average measure. We used the notation

(3) $$RMS\left(R,t\right)=\frac{1}{N}\sum_{i=1}^{N}∥Rx_{i}+t-y_{j^{*}}{∥}^{2}=\frac{1}{N}E\left(R,t\right),$$

for the evaluation of various methods in experiments.

In this paper we also define the registration problem with X, Y, R and t. The proposed method is applied to the rough alignment stage of the whole registration process, so that global measure is selected in consideration of convergence region, noise sensitivity and robustness. The histogram is brought in as a global feature notation to simplify the local features of the point set, as the notation H(X) denotes the feature histogram of *data* point cloud. As correlation-based method is demonstrated to outperform ICP algorithm [32], we defined the objective function to minimize the following cost function,

(4) COST(X,Y,R,t)=−Corr(H(X),H(T(Y,R,t)),

where Corr(·) is the correlation function. Since two point clouds are resembled, t is the difference of the centroids, which are easily calculated with the coordinates of the points. Ignoring the slight error of t, the problem is transformed into solving the value of R.

## 4. HEALPix-IA Algorithm

We call the algorithm HEALPix-IA because it is an initial alignment method based on HEALPix. To minimize the cost function in Equation (4), several steps need to be followed in order. In this section, HEALPix is introduced to reduce the dimensions by mapping the local features to 2-sphere first. After getting the histogram, corresponding points are estimated and optimized. The rotation can be estimated with the corresponding points and optimized with the correlation function.

### 4.1. Pixelization, Projection and Indexing

HEALPix (Hierarchical Equal Area isoLatitude Pixelization of a 2-sphere) is an algorithm for pixelization of the 2-sphere, and the associated class of map projections [35].

**Figure 1:**
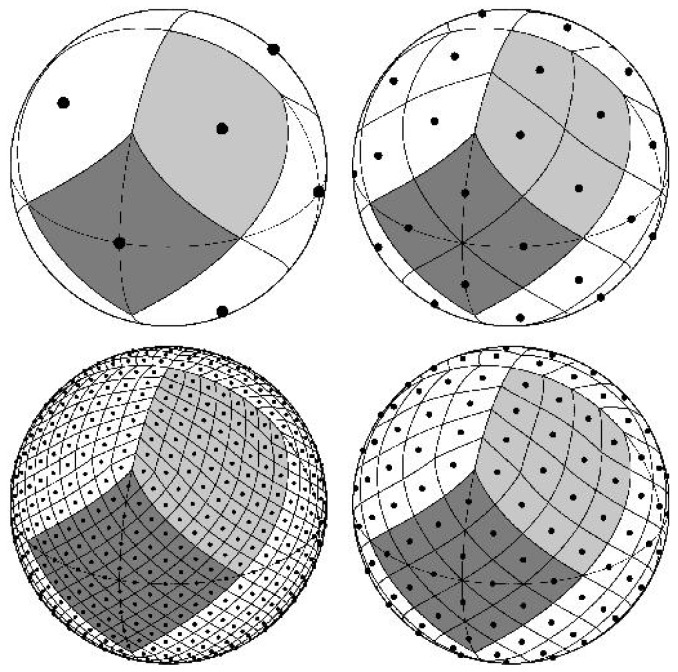
HEALPix: The resolution increase by three steps from the base level (the figure is cited from https://healpix.jpl.nasa.gov/).

It was first used for satellite missions to measure the cosmic microwave background anisotropy. The HEALPix map was created for the construction of full-sky maps of the microwave sky at an angular resolution of a few arcminutes. The sphere is hierarchically tessellated into curvilinear quadrilaterals. The lowest resolution partition is comprised of 12 base pixels. Resolution of the tessellation increases by division of each pixel into four new ones. Figure 1 illustrates (clockwise from upper-left to bottom-left) the resolution increase by three steps from the base level (i.e., the sphere is partitioned, respectively, into 12, 48, 192, and 768 pixels). We use the notation

(5) $$m=12\times 4^{n-1},n=1,2,3,\ldots ,$$

where *m* denotes the number of the pixel under *n*th level of the division.

The scheme has three advantages for our method: (1) Equal-area mapping can reduce the normal feature error generated in different posture (SO(3)). (2) Areas of all pixels at a given resolution are identical. (3) It has a less complexity computation of integrals over individual spherical harmonics. This gives a strong base for high speed performance of the proposed algorithm.

For the point sets *data* and *model*, let the scale be equalled by voxel filter to simplify the projection. In consideration of the efficiency and accuracy, let n=5 and the tessellation be divided into 3072 grids. As we obtain the EGI or normal sphere (NS) of the point cloud, it can be projected to HEALPix sphere (HS). The HealpixLib is utilized to generate sampling points and convert them from Spherical Coordination to Cartesian Coordination. The projection is equivalent to finding the nearest sample point of the normal vector using k-nearest neighbors algorithm. Thus, the projection from a single normal to the corresponding Healpix sample point is defined as

(6) $$P_{x}=P\left(N_{s}\right).$$

In this paper, we give hierarchic projection method to accelerate accessing data, which is different from other EGI methods. Let n=1 in Equation (5); we call the 12 pixels *basic girds*. In every basic grid, there are 256 *subdivided grids*. Here, we create the index using the notation

(7) $$H^{S}=\left\{H_{1}^{S},\ldots ,H_{i}^{S},\ldots ,H_{12}^{S}\right\},H^{T}=\left\{H_{1}^{T},\ldots ,H_{i}^{T},\ldots ,H_{12}^{S}\right\},$$

where $H_{i}^{S}$ and $H_{i}^{T}$ are the histograms of the 12 basic grids. Each histogram has 256 values, which stand for the intensity of subdivided grids. The mapping relation from the normals to histogram is defined as

(8) $$H_{x}=H\left(N_{s}\right),$$

where $N_{s}$ denotes a group normals and $H_{x}$ denotes the intensive histogram of the corresponding Healpix grids.

### 4.2. Point Correspondence Searching

Corresponding points are used to estimate the rotation. Generally, we use the peaks in the histogram to estimate at least two corresponding points. However, two situations would make this method fail. One is the histogram is relatively even in some range. The other is the two corresponding points are opposite (nearly 180 degree) in EGI. To tackle this problem, we use a relative feature instead of the absolute coordinate of the corresponding points.

First, finding the maximal grid in $H_{i}^{S}$ and $H_{i}^{T}$, we get

(9) $$P^{S}=\left\{P_{1}^{S},\ldots ,P_{i}^{S},\ldots ,P_{12}^{S}\right\},and\;P^{T}=\left\{P_{1}^{T},\ldots ,P_{i}^{T},\ldots ,P_{12}^{S}\right\},$$

where $P_{i}^{S}$ and $P_{i}^{T}$ are the indices of the maximal intensity and sorted in intensity descending order. Then, we let the center point of the sphere be the origin and get the vectors $V_{i}^{S}$, $V_{j}^{S}$, $V_{i}^{T}$ and $V_{j}^{T}$ point to the sample point of $P_{i}^{S}$, $P_{j}^{S}$, $P_{i}^{T}$ and $P_{j}^{T}$, where $P_{j}^{S}$ and $P_{j}^{T}$ are the indices of the maximal intensity in $H_{j}^{S}$ and $H_{j}^{T}$. For $P_{i}^{S}$, $P_{j}^{S}$ must meet the condition

(10) $$\arccos(V_{i}^{S},V_{j}^{S})<0.94,\;i<j⩽12,$$

or $P_{j}^{S}$ is excluded. The parameter 0.94 is an empirical value less than 1, as the two vectors are not absolutely coincident. The same condition is met with $V_{i}^{T}$ and $V_{j}^{T}$. Distance between the rest grids is calculated and denote as

(11) $$D^{S}=\left\{d_{ij}^{S}|P_{i}^{S},P_{j}^{S}\in P^{S}\right\},and\;D^{T}=\left\{d_{ij}^{T}|P_{i}^{T},P_{j}^{T}\in P^{T}\right\},$$

where $d_{ij}^{S}$ and $d_{mn}^{T}$ are the distance between each two rest grids. If ∃i,j, let

(12) $$\varepsilon =|d_{ij}^{S}-d_{ij}^{T}|<0.05,$$

then subdivided grids $P_{i}^{S}$ and $P_{j}^{S}$ can be aligned with $P_{i}^{T}$ and $P_{j}^{T}$ initially. The error 0.05 is an empirical value according to the difference and accuracy of the two data.

### 4.3. Point Correspondence Optimization

The Healpix method is limited by its accuracy for the pixelization. Although the sphere is divided into over 3000 grids, there is still intolerable error up to four degrees. To improve the corresponding point alignment accuracy, we optimize the method to calculate the peak of the intensive region in geometry instead of the sample points of Healpix grids.

In the last subsection, the two pairs of corresponding points are $P_{1}^{S}$, $P_{2}^{S}$, $P_{1}^{T}$ and $P_{2}^{T}$. The normal set is defined as

(13) $$N_{p}=\left\{N_{q}|P\left(N_{q}\right)\in \left\{P_{0},\ldots ,P_{n}\right\}\right\}$$

where $P_{n}$ are the n neighbours of the subdivided Healpix grid $P_{0}$. $P_{0}$ is one of the corresponding points mentioned above. Then, a plane *A* is created through the origin of the Healpix sphere, perpendicular to the normal vector of $P_{0}$. The projection of $N_{p}$ is defined on the plane *A*, as $N_{p}^{\prime }\in R^{2}$. In this optimal method, the intensity peak in plane *A* is searched by KD-tree using a circle with fixed radius, which is denoted as

(14) $$r=\frac{\max∥x_{i}-x_{j}∥}{\tau },$$

where τ is the parameter to control radius, and numerator is the range of the normals in *x* axis. In the range of radius *r*, we mesh the region with grid in fixed step, and search for the maximum intensity grid with KD-tree method. Assuming $\left(x_{p},y_{p}\right)$ is the peak point, it is projected back to Healpix sphere, denoted as $P_{0}^{\prime }$, which is the modified corresponding point. Thus, we modify the corresponding points as ${P_{1}^{S}}^{\prime }$, ${P_{2}^{S}}^{\prime }$, ${P_{1}^{T}}^{\prime }$ and ${P_{2}^{T}}^{\prime }$. Algorithm 1 describes how to estimate and optimize the corresponding points.

### 4.4. Rotation Estimation

To align two EGIs, we assume that the origins coincide, so that only two rotation angles are needed for entire alignment. Defining the normal vectors of the corresponding points ${V_{i}^{S}}^{\prime }$, and ${V_{i}^{T}}^{\prime }$, the rotation vector is

(15) $$V_{i}=\frac{{V_{i}^{T}}^{\prime }\times {V_{i}^{S}}^{\prime }}{∥{V_{i}^{T}}^{\prime }\times {V_{i}^{S}}^{\prime }∥},$$

and rotation angle is

(16) $$\theta_{i}=\frac{\arccos({V_{i}^{T}}^{\prime }\times {V_{i}^{S}}^{\prime })}{∥{V_{i}^{T}}^{\prime }∥\cdot ∥{V_{i}^{S}}^{\prime }∥}.$$

According to Rodrigues’ rotation formula, the rotation matrix is

(17) $$R_{i}=\cos\left(\theta_{i}\right)I+\left(1-\cos\left(\theta_{i}\right)\right)V_{i}V_{i}^{H}+\sin\left(\theta_{i}\right)\left[\begin{array}{ccc} 0 & -V_{iz} & V_{iz}\\ V_{iz} & 0 & -V_{ix}\\ -V_{iy} & V_{ix} & 0, \end{array}\right]$$

where *I* is identity matrix, $V_{i}^{H}$ is the transposed matrix, and $V_{ix}$, $V_{iy}$, and $V_{iz}$ are the components of $V_{i}$. Then, we calculate the final rotation matrix with Equation (17),

(18) $$R=R_{1}\cdot R_{2}.$$

**Algorithm 1:** Estimate and optimize the corresponding points.**input**: *ModelS* and *DataT* with normals of size N×6 (*N* is the scale of *Model*/*Data*)**output**:  2 pairs of corresponding points ${P_{1}^{S}}^{\prime }$, ${P_{2}^{S}}^{\prime }$, ${P_{1}^{T}}^{\prime }$ and ${P_{2}^{T}}^{\prime }$
**1**

extract normals $N_{S}$ and $N_{T}$ from *S* and *T*

**2**
$H^{S}\leftarrow H\left(N_{S}\right)$; $H^{T}\leftarrow H\left(N_{T}\right)$;
// see Equations (7) & (8)

**3**
**for**i←1**to** 12 **do**
**4**
 $H_{S}\left(i\right),I_{S}\left(i\right)\leftarrow \max\left(H_{i}^{S}\right)$;
// maximum intensity and the index

**5**
 $H_{T}\left(i\right),I_{T}\left(i\right)\leftarrow \max\left(H_{i}^{T}\right)$
**6**
 $N_{S}\left(i\right)\leftarrow I_{S}\left(i\right)$; $N_{T}\left(i\right)\leftarrow I_{T}\left(i\right)$;
// normals of the max

**7**
 $P_{i}^{S}\leftarrow P\left(N_{S}\left(i\right)\right)$; $P_{i}^{T}\leftarrow P\left(N_{T}\left(i\right)\right)$;
// see Equation (6)

**8**
 construct Struct $Q\left(i\right)=\{H\left(i\right),I\left(i\right),P_{i}\}$
**9**

**end for**

**10**
$Q_{S}\leftarrow$sortrows ($H_{S}$; $Q_{T}\leftarrow$) sortrows ($H_{T}$);
// descending sort

**11**

i=2

**12**

$P_{temp}^{S}=P_{2}^{S}$

**13**

**while**
$\arccos(V_{i}^{S},V_{temp}^{S})>0.94$
**do**

// see Eqution (10)

**14**
 $P_{temp}^{S}=P_{i+1}^{S}$
**15**
 i←i+1
**16**

**end while**

**17**

$P_{2}^{S}=P_{temp}^{S}$

**18**
**for**i←2**to** 12 **do**
**19**
 $P_{temp}^{T}=P_{i}^{T}$
**20**
 **if**
$|d_{1temp}^{S}-d_{1temp}^{T}|<0.05$
**then**
// see Equation (12)

**21**
  $P_{2}^{T}=P_{temp}^{T}$
**22**
  break;
**23**
 **end if**
**24**

**end for**

**25**
let n=4 then calculate $N_{1}^{S},N_{2}^{S},N_{1}^{T},N_{2}^{T}$;
// see Equation (13)

**26**

$\left[a,b,c\right]=V_{1}^{S}$

**27**
create $e_{1}=\left(1,1,-\frac{a+b}{c}\right)$ in plane *A* then $e_{2}=e_{1}\times V_{1}^{S}$
**28**
${N_{1}^{S}}^{\prime }={\left(\left[e_{i};e_{2}\right]\cdot {\left(N_{1}^{S}\right)}^{T}\right)}^{T}$;
// project normals to A

**29**
calculate $r_{1}^{S}$;
// see Equation (14)

**30**
 mesh $r_{1}^{S}\times r_{1}^{S}$ region into 100 grids;
**31**
search $\left(x_{p},y_{p}\right)$ with the peak intensity in meshed grids;
**32**
calculate inverse projection point ${P_{1}^{S}}^{\prime }$
**33**
calculate ${P_{2}^{S}}^{\prime }$, ${P_{1}^{T}}^{\prime }$, ${P_{2}^{T}}^{\prime }$

### 4.5. Correlation Searching

According to Equation (4), cost function is employed to maximize the correlation of the *model* and transformed *data*. In this method, we use cross correlation method and simply define the correlation function as

(19) $$Corr=\frac{\langle H^{S},H^{T}\rangle }{∥H^{S}∥\cdot ∥H^{T}∥}.$$

When Corr→1, the *model* and transformed *data* are most correlative. Iterative search is carried out with halt conditions in this method. Moreover, parameter *correlation* and *iteration* are recommended as 0.98 and 50.

In the process of the optimizing search, random rotation angle θ is given in rotation matrix form

(20) $$T_{rand}=R_{\omega }\left(\theta \right)R_{\phi }\left(\theta \right)R_{\kappa }\left(\theta \right),$$

where ω, ϕ, κ∈(−π,π). The basic rotations follow the right-hand rule. Algorithm 2 describes the search strategy of transformation estimation.

**Algorithm 2:** Optimal transformation search.**input**: *ModelS* and *DataT* with normals of size N×6 (*N* is the scale of *Model*/*Data*)**output**: transformation *T*, correlation corr, iteration iter
**1**
initialize iter=0;stall=0;corr=0;T=eye(3)

**2**
extract normals $N_{S}$ and $N_{T}$ from *S* and *T*
**3**
$H^{S}\leftarrow H\left(N_{S}\right)$; 
// see Equation (8)

**4**

**while**
*corr<0.98 and iter<50*
**do**

**5**
 estimate initial transformation $T_{temp}$ with Equations (15)∼(18)
**6**
 $H^{T}\leftarrow H\left({\left(T_{temp}\cdot {\left(N_{T}\right)}^{T}\right)}^{T}\right)$
**7**
 calculate $corr_{temp}$;
// see Equation (19)

**8**
 stall←stall+1
**9**
 **if**$corr<corr_{temp}$
**then**

**10**
  **if**
$corr_{temp}>corr\times 1.005$
**then**
**11**
   stall←stall−1
**12**
  **end if**
**13**
  $corr\leftarrow corr_{temp}$
**14**
  $T\leftarrow T_{temp}\cdot T$
**15**
  $N_{T}\leftarrow {\left(T_{temp}\cdot {\left(N_{T}\right)}^{T}\right)}^{T}$
**16**
 **end if**
**17**
 **if**
*stall⩾3 and $corr_{temp}<0.98$*
**then**
**18**
  $T\leftarrow T_{rand}\cdot T$; 
// see Equation (20)

**19**
  $N_{T}\leftarrow {\left(T_{rand}\cdot {\left(N_{T}\right)}^{T}\right)}^{T}$
**20**
  stall←0
**21**
 **end if**
**22**
 iter←iter+1
**23**

**end while**


## 5. Results and Evaluation

The proposed algorithm was verified with both model simulation and real data. It was compared with other EGI-based methods and other sophisticated rough registration algorithms.

### 5.1. Simulation

**Figure 2:**
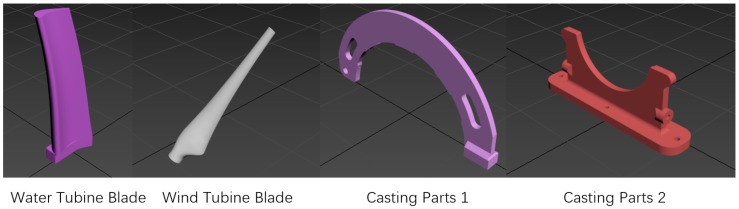
CAD models for HEALPix-IA tests.

Three tests were carried out in simulation. The test method was self-registration with random rotations. Different division methods and point correspondence optimization were verified in the first test. The performance of the proposed algorithm was compared with other rough registration methods in the second test. How different features affect on the method was tested in the end. The four CAD models shown in Figure 2 were prepared to test HEALPix-IA algorithm. They were all casting parts that need grinding after molding.

#### 5.1.1. Compared with EGI-Based Methods

**Figure 3:**
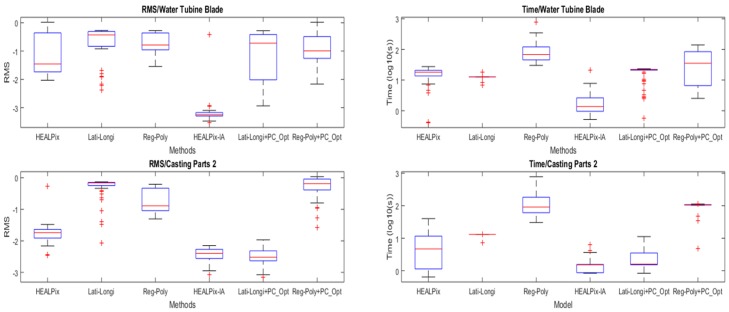
The results of different EGI-based methods for HEALPix-IA tests.

In the proposed algorithm, new division method and point correspondence method are suggested. To measure the effectivity of the algorithm, root mean square distances (RMS) and running time are two key evaluation criteria. We used several different types of models to test the registration results with two EGI-based algorithms. Latitude and longitude tessellation (Lati-Longi, in Ameesh’s algorithm [21]) and regular polyhedron tessellation (Reg-Poly, in Christoph’s algorithm [22]) were both tested in this experiment. In addition, point correspondence optimization (PC-Opt) was also separated. Six trials included HEALPix tessellation, Lati-Longi tessellation, Reg-Poly tessellation, HEALPix-IA, and Lati-Longi, respectively, with point correspondence optimization and Reg-Poly with point correspondence optimization, carried out with two models and 50 samples. The results are shown in Figure 3, where RMS and running time are both in logarithm. The tests were configured with the same halt conditions: 0.02 correlation value and 50 iterations.

The two selected models were water turbine blade and casting parts 2 with typical features friendly to EGI-based methods. The first three groups without PC-Opt in the boxplot show HEALPix division method won in both RMS and running time, while the three groups with PC-Opt performed more distinctly. The comparison between each division method with or without PC-Opt showed that PC-Opt gave better performance for HEALPix and Lati-Longi. HEALPix and Lati-Longi overwhelmed Reg-Poly in running time because the letter one uses an iteration division method that costs time for every generation. Furthermore, the higher level the sphere is divided, the more time is cost. This is the main limit of the regular polyhedron tessellation. The methods with PC-Opt ran more accurately than the ones with only divide tessellations. With the same RMS for two methods with PC-Opt, the running time of the proposed algorithm had an advantage over other methods.

#### 5.1.2. Compared with Other Rough Registration Methods

**Figure 4:**
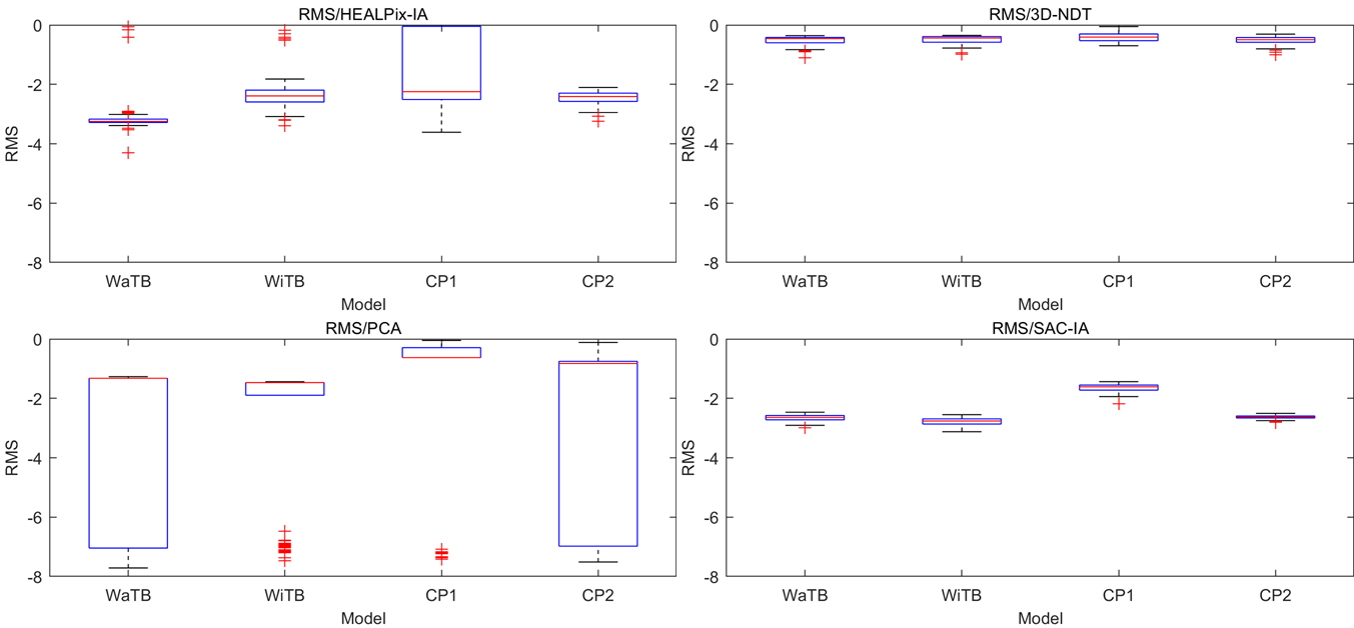
RMS results in logarithm of four rough registration methods contrast tests.

**Figure 5:**
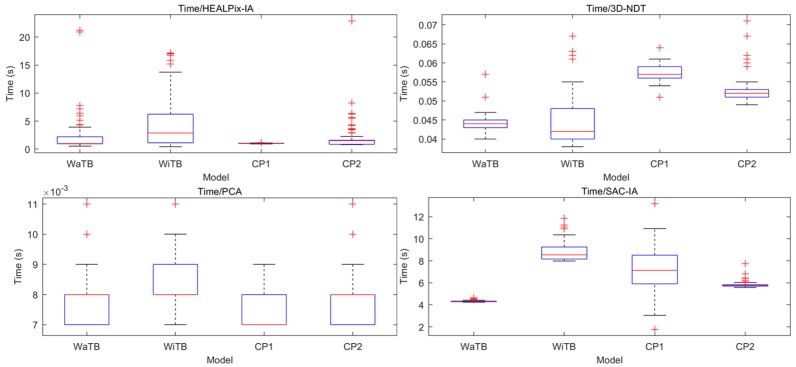
Running time results of four rough registration methods contrast tests.

With PCL library, PCA, 3D-NDT, and Sac-IA were applied, respectively, on the registration tasks of four CAD models together with HEALPix-IA (C + + ). One hundred trials were carried out for each model. The results are shown in Figure 4 and Figure 5. The RMS values are shown in logarithm.

From the boxplot, we can see HEALPix-IA performed the best in both accuracy and efficiency and PCA performed the worst. For accuracy, HEALPix-IA and Sac-IA obtained about the same RMS; however, Sac-IA had a more stable performance because it had over 1000 searching iterations, which resulted in long running time. In addition, HEALPix-IA won by almost an order of magnitude in running time over Sac-IA. Nevertheless, 3D-NDT’s results depended on its complex parameter configurations, which would hinder its application in automatic process. Although it ran quickly, the accuracy was quite low. Furthermore, 3D-NDT is not safe because it is a local method that is easily trapped into local minima. The three other global methods are better choices to carry out an automatic rough registration task.

#### 5.1.3. Tests on Real Data with Different Features via Various Sensors

**Figure 6:**
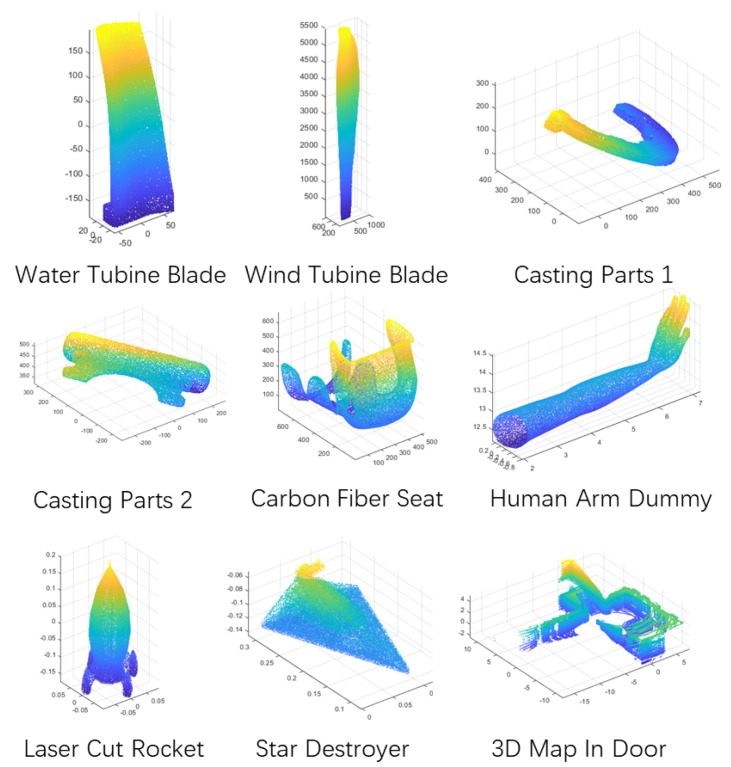
Other types of models for HEALPix-IA tests.

**Figure 7:**
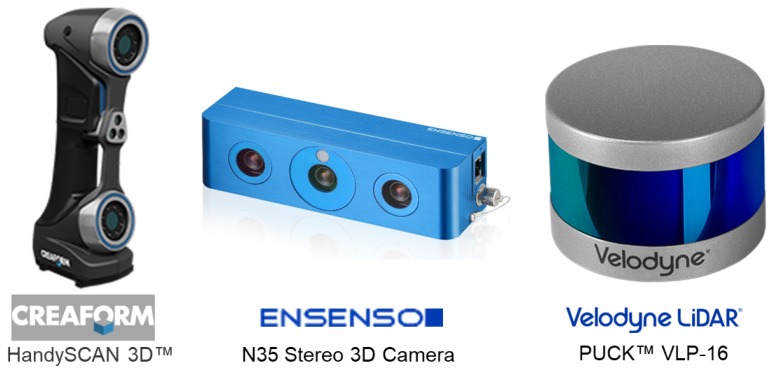
Various sensors used for tests.

**Figure 8:**
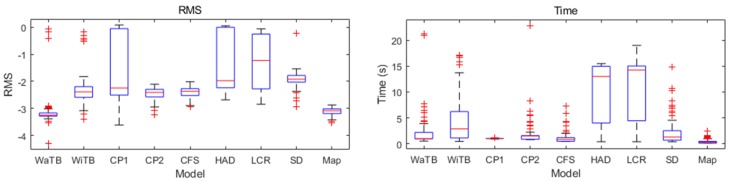
Results of different types of models for HEALPix-IA tests.

We chose nine different data, shown in Figure 6, to test the proposed algorithm. Three kinds of sensors were used in this test: 3D laser scanner, 3D cameras and 3D lidar (Figure 7). The 3D laser scanner can reach the volumetric accuracy up to 30 μm and the 3D camera with image stitching technique can reach about 0.8 mm. All data obtained from the sensors had their noise eliminated. The different features are explained in Table 1 and the test results are shown in Figure 8.

In this test, we raised the search iteration to 50 generations to adapt to different types of features. The boxplot shows that HEALPix-IA performed poorly with “human arm dummy” and “laser cut rocket”. The reason is that they both have the constant EGI features similar to sphere surface, which is a disadvantage of all EGI-based methods. The “casting parts 1” with thin-walled plane led to a fast convergence in searching iteration, so that it performed a quick registration with unstable accuracy. Besides, the two other sensors achieved better results in both accuracy and efficiency. In summary, HEALPix-IA is applicable with most features and different sensors, yet its stability needs to be improved further.

### 5.2. MA Analysis Test

**Figure 9:**
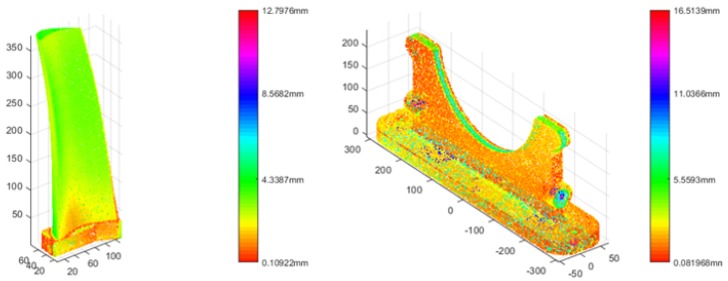
Results of real workpiece MA analysis using HEALPix-IA.

In this paper, we suggest HEALPix-IA is applicable to MA analysis for manufacturing. In this experiment, two real workpieces were scanned by 3D scanner (Creaform HandyScan 700) and aligned to the CAD models. As the rough registration is the first step for MA analysis, another evaluation criterion, optimization time for MA analysis process, was used. The results are shown in Table 2 with Sac-IA and HEALPix-IA. Here, we abandoned PCA and 3D-NDT for stability and local method. HEALPix-IA was verified to perform relatively better in accuracy (RMS) and efficiency (time) for MA analysis application with almost the same stability though the variance values. The MA analysis results are shown in Figure 9 which can be used for follow-up manufacturing processes such as debugging and grinding.

## 6. Conclusions

In this paper, a new rough registration method—HEALPix-IA—is proposed especially for MA analysis application. HEALPix-IA is based on EGI, and has the same features with other EGI-based algorithms, such as global method, robust to noise, and sensitive to constant EGI. Furthermore, we suggest a new division method, which was proven to be more effective. Point correspondence optimization is also implemented to improve the accuracy. HEALPix-IA was compared with other sophisticated rough registration algorithms, PCA, 3D-NDT and Sac-IA, on RMS and running time, utilizing nine real scanned data and two pairs of datasets for MA analysis, which were obtained from three different kinds of sensors. The experiments verified HEALPix-IA shows a better performance on accuracy and efficiency.

## Figures and Tables

**Table 1:** Features of nine data for tests.

Model (Abbreviation)	Features	Sensors
Water Turbine Blade (WaTB)	complex Curve Surface	3D laser scanner
Wind Turbine Blade (WiTB)	complex curve Surface	3D laser scanner
Casting Parts 1 (CP1)	thin-walled, large plane	3D laser scanner
Casting Parts 2 (CP2)	mirror symmetry	3D laser scanner
Carbon Fiber Seat (CFS)	thin-walled, curve surface	3D laser scanner
Human Arm Dummy (HAD)	unstructureed features	3D laser scanner
Laser Cut Rocket (LCR)	rotational symmetry	3D laser scanner
Star Destroyer (SD)	mirror symmetry	3D Camera
3D Map In Door (Map)	low dense data	3D LiDAR

**Table 2:** MA analysis with different rough registration methods.

Models and Criteria		Sac-IA	HEALPix-IA
WaTB-RMS	Ave	2.9 × 10^−2^	3.4 × 10^−3^
	Var	8.1 × 10^−4^	4.9 × 10^−5^
WaTB-time (ms)	Ave	4932.1	3830.8
	Var	6.3 × 10^3^	2.7 × 10^4^
WaTB-opt time (ms)	Ave	7222.7	10,110.5
	Var	1.6 × 10^7^	2.2 × 10^7^
CP2-RMS	Ave	1.2 × 10^−2^	7.2 × 10^−3^
	Var	3.5 × 10^−5^	1.1 × 10^−6^
CP2-time (ms)	Ave	5953.2	4818.3
	Var	8.7 × 10^3^	4.5 × 10^3^
CP2-opt time (ms)	Ave	9049.9	8066.5
	Var	2.65 × 10^7^	1.4 × 10^7^

## Abbreviations

The following abbreviations are used in this manuscript:

ICP	Iterative Closest Point
EGI	Extended Gaussian Image
SLAM	Simultaneous Localization And Mapping
MA	Machining Allowance
CMM	coordinate measuring machine
PCA	principal component analysis
FPFH	Fast Point Feature Histograms
Sac-IA	Sample Consensus-Initial Alignment
NDT	Normal Distributions Transform
RMS	Root Mean Square distances
